# Learning the structure of gene regulatory networks from time series gene expression data

**DOI:** 10.1186/1471-2164-12-S5-S13

**Published:** 2011-12-23

**Authors:** Haoni Li, Nan Wang, Ping Gong, Edward J Perkins, Chaoyang Zhang

**Affiliations:** 1 School of Computing, University of Southern Mississippi, Hattiesburg, MS 39406, USA; 2 Environmental Services, SpecPro Inc, San Antonio, TX 78216, USA; 3 Environmental Laboratory, U.S. Army Engineer Research and Development Center, Vicksburg, MS 39180, USA

## Abstract

**Background:**

Dynamic Bayesian Network (DBN) is an approach widely used for reconstruction of gene regulatory networks from time-series microarray data. Its performance in network reconstruction depends on a structure learning algorithm. REVEAL (REVerse Engineering ALgorithm) is one of the algorithms implemented for learning DBN structure and used to reconstruct gene regulatory networks (GRN). However, the two-stage temporal Bayes network (2TBN) structure of DBN that specifies correlation between time slices cannot be obtained by score metrics used in REVEAL.

**Methods:**

In this paper, we study a more sophisticated score function for DBN first proposed by Nir Friedman for stationary DBNs structure learning of both initial and transition networks but has not yet been used for reconstruction of GRNs. We implemented Friedman's Bayesian Information Criterion (BIC) score function, modified K2 algorithm to learn Dynamic Bayesian Network structure with the score function and tested the performance of the algorithm for GRN reconstruction with synthetic time series gene expression data generated by GeneNetWeaver and real yeast benchmark experiment data.

**Results:**

We implemented an algorithm for DBN structure learning with Friedman's score function, tested it on reconstruction of both synthetic networks and real yeast networks and compared it with REVEAL in the absence or presence of preprocessed network generated by Zou&Conzen's algorithm. By introducing a stationary correlation between two consecutive time slices, Friedman's score function showed a higher precision and recall than the naive REVEAL algorithm.

**Conclusions:**

Friedman's score metrics for DBN can be used to reconstruct transition networks and has a great potential to improve the accuracy of gene regulatory network structure prediction with time series gene expression datasets.

## Background

High-content technologies such as DNA microarrays can provide a system-scale overview of how genes interact with each other in a network context. This network is called a gene regulatory network (GRN) and can be defined as a mixed graph over a set of nodes (corresponding to genes or gene activities) with directed or undirected edges (representing causal interactions or associations between gene activities) [1]. Various mathematical methods and computational approaches have been proposed to reconstruct GRNs, including Boolean networks [2], information theory [3,4], differential equations [5] and Bayesian networks [6-8]. GRN reconstruction faces huge intrinsic challenges on both experimental and theoretical fronts, because the inputs and outputs of the molecular processes are unclear and the underlying principles are unknown or too complex. In the previous work, we compared two important computational approaches, Dynamic Bayesian networks (DBNs) and Probabilistic Boolean networks for reconstructing GRNs using a time-series dataset from the Drosophila Interaction Database, and found that DBN outperforms PBN [9]. In this paper, we emphasize the DBN approach.

Dynamic Bayesian networks (DBNs) are belief networks that represent the stochastic process of a set of random variables over time. The hidden Markov model (HMM) and the Kalman filter can be considered as the simplest DBNs. However, Kalman filters can only handle unimodal posterior distributions and linear models, whereas parameterization of HMM grows exponentially with the number of state variables [10]. Several algorithms have been developed to learn structure for belief networks from both complete [6,10-12] (without missing values) and incomplete [13,14] (with missing values) datasets. Structure Expectation-Maximization (SEM) has been developed for learning Probabilistic network structure from data with hidden variables and missing values [13]. A structure learning algorithm has also been developed for high-order and non-stationary dynamic probabilistic models [15].

A commonly used structure learning algorithm is based on REVEAL (REVerse Engineering ALgorithm) [6,12] which learns the optimal set of parents for each node of a network independently, based on an information theoretic concept of mutual information analysis. However, the two-stage temporal Bayes network (2TBN) cannot be well recovered by application of REVEAL. In this work, we implemented a more sophisticated algorithm, proposed by Friedman [10], to learn the structure of both initial networks and transition networks, which specified a stationary correlation between two consecutive time periods. Compared with Murphy's algorithm, it improves performance in two ways. First, in score function, it considers time lags that may happen in biological processes. Second, it fetches the relationship which gains the maximum score function in the same time period or in the two consecutive time periods. Thus, Friedman's DBN structure learning algorithm was used in our work and its performance in terms of reconstruction accuracy was also evaluated using synthetic gene expression datasets and a real yeast time-series benchmark dataset.

In the following sections, we first provide an introduction to DBN and existing DBN algorithms for reconstruction of GRNs. We then present an implementation of Friedman's DBN algorithm. Finally, we apply the algorithms to synthetic datasets and a real yeast benchmark dataset, and compare its performance to the commonly used Murphy's DBN algorithm [12,16] based on REVEAL.

## Methods

### Dynamic Bayesian networks

**Figure 1:**
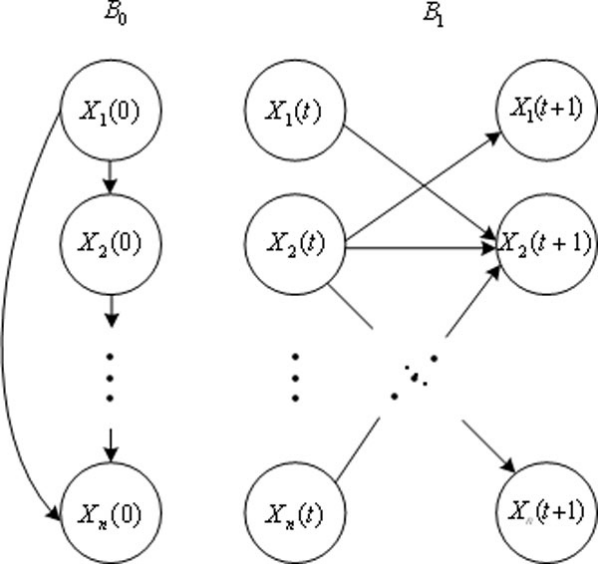
**The basic building block of DBN**.

A DBN is a probabilistic network defined as a pair (*B*_0_,*B*_→_) representing the joint probability distribution over all possible time series of variables *X*={*X*_1_,*X*_2,...,_*X_n_*}, where *X_i _*represents the discretized-valued random variables in the network. *X_i _*is composed of an initial state of Bayesian network *B*_0_=(*G*_0_,Θ_0_)and a transition Bayesian network *B_→_*= (*G*_→_,Θ_→_). In time slice 0, the parents of *X_i_*_[0] _are specified in the prior network *B*_0_, the parents of *X_i_*_[t+1] _are those specified in time slice *t *and *t*+1 in *B*_→_. The structure of a two-stage temporal Bayes network (2TBN) is showed in Figure 1. DBN theory is generally based on two assumptions. First, the process is *Markovian *in *X*, i.e. *P*(*X*_[*t*+1]_|*X*_[0]_,...,*X*[*t*])=*P*(*X*_[*t*+1]_|*X*_[*t*]_). The other assumption is that the process is stationary, i.e. the transition probability *P*(*X*_[*t*+1]_|*X*_[*t*]_) is independent of *t *.

### Bayesian information criterion for DBN

Given a Bayesian network with structure *G*, parameters and the observations *D*, we define a structure prior that implies a prior probability on different graph structures, and a parameter prior, that puts a probability on different choice of parameters once the graph is given. By Bayes rule,

$$P\left(G|D\right)=\frac{P\left(D|G\right)P\left(G\right)}{P\left(D\right)}$$

where the denominator is simply a normalized factor. Thus, we define the Bayesian score as:

$$score_{B}\left(G:D\right)=logP\left(D|G\right)+logP\left(G\right)$$

where

$$P\left(D|G\right)=\int_{\theta_{G}}P\left(D|\theta_{G},G\right)P\left(\theta_{G}|G\right)d\theta_{G}$$

where P(D|θ_G_,G) is the marginal likelihood of the data given the network 〈G,θ_G_〉 and P(θ_G_|G) is our pior.

Under Dirichlet distribution prior for all parameters in the network, when *M*→∞, we have

(1) $$logP\left(D|G\right)=l\left(\theta_{G}:D\right)-\frac{logM}{2}Dim\left[G\right]+O$$

where [*G*] is the model dimension, or the number of independent parameters in *G*.

This approximation is called the Bayesian information criterion (BIC). N. Friedman, et al. deduce BIC for Dynamic Bayesian Network in his work, which is briefly described below.

It is assumed that the dataset *D *is composed of *N_seq _*complete observations. The first such sequence has length *N_i _*and specifies values for the variables *x_l_*[0],...,*x_l_*[*N_l_*], which means in different time slice the number of observations can be different. With such a dataset, we can learn *B*_0 _from *N_seq _*observations of initial slice, and learn *B*_→ _by $N=\sum_{l}N_{l}$ transactions of transition slices.

We use the following notations,

$$\begin{array}{c} \theta_{i,j_{i}^{\prime },k_{i}^{\prime }}^{\left(0\right)}=\mathrm{Pr}\left(X_{i}\left[0\right]=\overset{\prime }{\underset{i}{k}}|Pa\left(X_{i}\left[0\right]\right)=\overset{\prime }{\underset{i}{j}}\right)\\ \theta_{i,j_{i}^{\prime },k_{i}^{\prime }}^{\to }=\mathrm{Pr}\left(X_{t}\left[t\right]=\overset{\prime }{\underset{i}{k}}|Pa\left(X_{i}\left[t\right]\right)=\overset{\prime }{\underset{i}{j}}\right)\\ N_{i,j_{i}^{\prime },k_{i}^{\prime }}^{\left(0\right)}=\sum_{l}I\left(X_{i}\left[0\right]=k_{i}^{\prime },Pa\left(X_{i}\left[0\right]\right)=j_{i}^{\prime };x^{l}\right)\\ N_{i,j_{i}^{\prime },k_{i}^{\prime }}^{\to }=\sum_{l}\sum_{t}I\left(X_{i}\left[t\right]=k_{i},Pa\left(X_{i}\left[t\right]\right)=j_{i}^{\prime };x^{l}\right) \end{array}$$

where *I*(·;*x^l^*) is an indicator function which equals 1 if the corresponding event occurs in sequence *x^l^*, and 0 otherwise.

The likelihood function decomposes as:

$$\mathrm{Pr}\left(D|G,\theta_{G}\right)=\prod_{i}\prod_{j_{i}^{\prime }}{\prod_{k_{i}^{\prime }}\left({\theta^{\left(0\right)}}_{i,j^{\prime },k^{\prime }}\right)}^{N_{i,j^{\prime },k^{\prime }}^{\left(0\right)}}\times \prod_{i}\prod_{j_{i}}{\prod_{k_{i}}\left({\theta^{\to }}_{i,j,k}\right)}^{N_{i,j^{\prime },k^{\prime }}^{\to }}$$

and the log-likelihood is given by

$$L\left(B:D\right)=\sum_{i}\sum_{j_{i}^{\prime }}\sum_{k_{i}^{\prime }}N_{i,j^{\prime },k^{\prime }}^{\left(0\right)}\log{\theta^{\left(0\right)}}_{i,j^{\prime },k^{\prime }}+\sum_{i}\sum_{j_{i}}\sum_{k_{i}}N_{i,j^{\prime },k^{\prime }}^{\to }\log{\theta^{\to }}_{i,j,k}$$

Such decomposition implies that *B*_0 _is independent from *B_→_*, so we can give BIC score as BIC(G:D) = BIC_0_+BIC→

where,

$$\begin{array}{c} BIC_{0}=\sum_{i}\sum_{j_{i}^{\prime }}\sum_{k_{i}^{\prime }}N_{i,j_{i}^{\prime },k_{i}^{\prime }}^{\left(0\right)}\log\theta_{i,j_{i}^{\prime },k_{i}^{\prime }}^{\left(0\right)}-\frac{\log N_{seq}}{2}\#G_{0}\\ BIC_{\to }=\sum_{i}\sum_{j_{i}}\sum_{k_{i}}N_{i,j_{i},k_{i}^{\prime }}^{\to }\log\theta_{i,j_{i},k_{i}}^{\to }-\frac{\log N}{2}\#G_{\to } \end{array}$$

### Learning network structure

**Figure 2:**
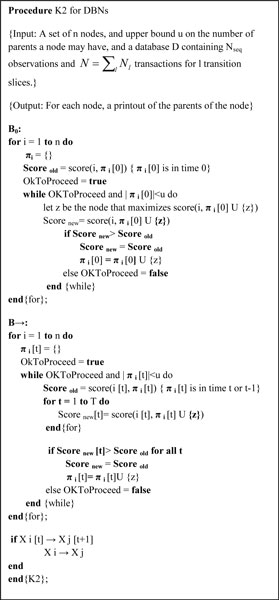
**Modified K2 algorithm for use in Friedman's algorithm on structure learning for dynamic Bayesian network (DBN)**.

Under Friedman score metrics, the maximized score can be exploited by any Bayesian structure learning procedure, **s**uch as hill-climbing search procedures. In this paper, we modify K2 algorithm, and adapt it to learn structure for DBN, as described in Figure 2. K2 algorithm was described by Gregory E. Cooper [11]. It begins by making the assumption that a node has no parents, and adds gradually with those that most increase the score of the structure. Different from the K2 Bayesian structure learning algorithm, an additional constrain must be imposed which is that the transition network structure must repeat between time slices over time. Furthermore, we learn best structure of *B*_0 _independently of that of *B*_→_. We find the maximum score function and add a correlation between the factors in consecutive time slices or the same time slice if the relationship increases the score function. We stop adding parents to the node, when the addition of no single parent can increase the score.

### Existing approaches for comparison

For convenience of performance analysis in the next section, we briefly describe Murphy and Zou's previous work here and present results in the next section. The widely used DBN implementation developed by Murphy and Mian (called Murphy's DBN hereafter) is based on REVEAL [12]. Given an unknown structure with full observations, the algorithm learns the parent set for each node independently. There are 2^n^ such sets, which can be arranged in a lattice for the permutation of factors. The problem is to find the highest score in the lattice. The approach taken by REVEAL is started from the bottom of the lattice, and evaluates the score at all points in the successive level until a point is found with a score of 1.0. Zou and Conzen [17] proposed a method to generate a preprocessed network for potential regulators by biological interpretation of time course microarray data. It assumes that the gene with earlier initial up-regulation is the potential regulator of those with later initial up-regulation. This preprocessed network is used to narrow down the search space for Murphy's DBN algorithm because it requires excessive time to find a permutation for each node even when imposing a maximum number of parents for the nodes if the network dimension is large.

## Results and discussion

The Friedman's algorithm described in the method section was implemented based on Murphy's BNT tool box (Bayes Net Toolbox for Matlab). We tested four cases of DBN algorithms on reconstruction of synthetic networks. The four methods are: (1) Zou's preprocessed networks consisting of potential regulators by biological interpretation of time course microarray data (Zou&Conzen), (2) Murphy's DBN, implemented in conjunction with the preprocessed networks (Kevin Murphy + Zou&Conzen), (3) Friedman's algorithm (Nir Friedman), and (4) Friedman's algorithm combined with the preprocessed networks (Friedman + Zou&Conzen).

Precision (P) and recall (R) were used as the metrics for performance comparison. Here, R is defined as C*_e_*/(C*_e_* + M*_e_*) and P is defined as C*_e_*/(C*_e_* + F*_e_*), where C*_e_* denotes true positive edges that exist in both the true network and the predicted network, M*_e_* false negative edges that exist in the true network but not in the inferred network, and F*_e_* false positive edges that do not exist in the true network but do exist in the predicted network.

### Synthetic data

The synthetic datasets and network were generated using GeneNetWeaver from DREAM (Dialogue for Reverse Engineering Assessments and Methods) projects [18]. We used sub-networks of different sizes (i.e., 10, 20, 50 and 100 genes) with randomly picked factors from a high-dimensional yeast GRN with 4441 nodes and 12873 edges. A model consisting of ordinary and stochastic differential equations and Gaussian noise model was used to generate synthetic gene expression data with a total of 21 time points and 10 replicates for each time slice.

**Figure 3:**
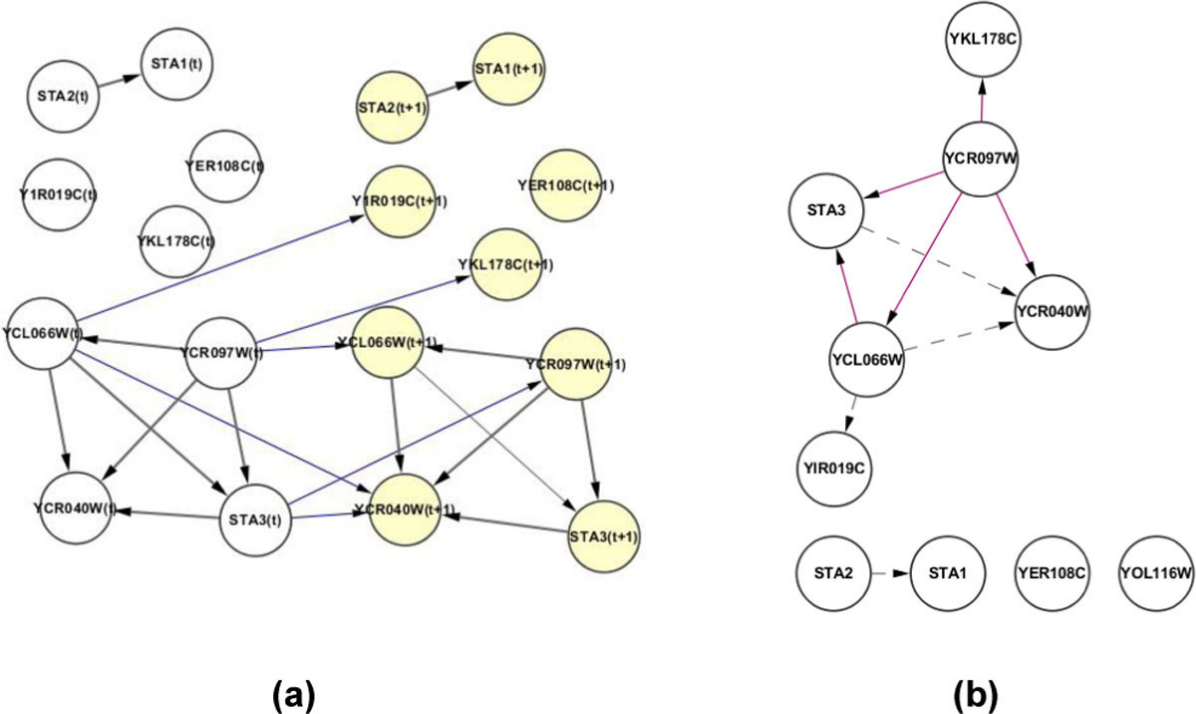
**(a) A transition network of 10 genes learned by Friedman score metrics**. The left column shows the genes at time t, and the right column the corresponding gene at the next time slice. (b) The gene regulatory network converted from (a).

An example of the 10-gene transition network reconstructed using Friedman's algorithm is shown in Figure 3a. This network was converted to a GRN (Figure 3b) by forming a relationship between two genes if the two are related in time t and time t+1 as the DBN theory suggests.

**Figure 4:**
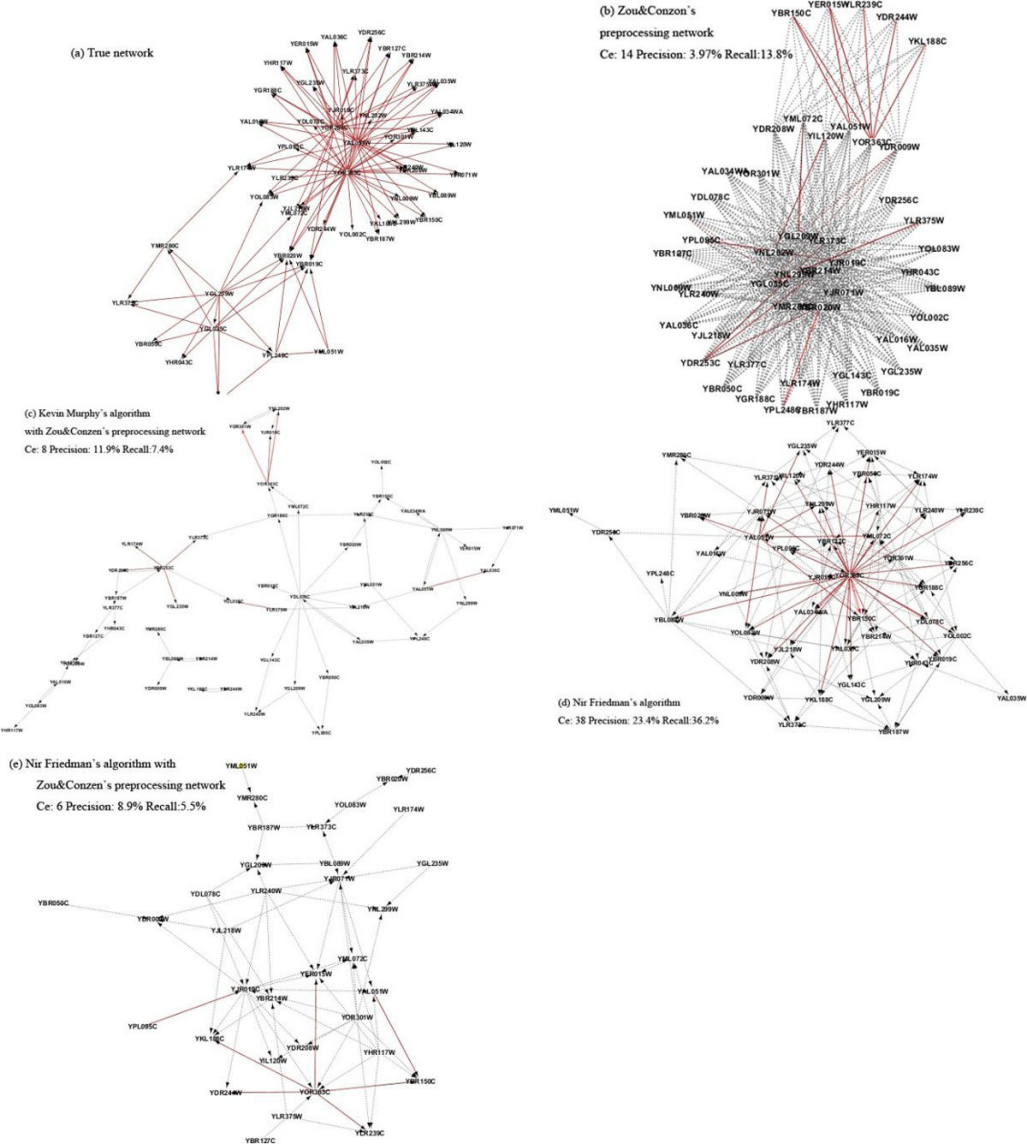
**The 50-gene network reconstructed by different algorithms with dashed lines indicating false positive edges, and solid lines true positive edges**. (a) The true network, (b) *Zou&Conzen's prior *network algorithm, (c) Murphy's algorithm, (d) Friedman's algorithm, (e) Friedman's algorithm combined with the prior network.

The second example is the GRNs with 50 genes as shown in Figure 4 where the dashed lines indicate false positive edges, and solid lines true positive edges. The true network used to generate synthetic data in GeneNetWeave is given in Figure 4(a). The preprocessed network includes a large number of false positive edges (dashed lines), resulting in a lower accuracy. The GRN reconstructed by Murphy& Zou, as given in Figure 4(c), is a sparse network that has a lower recall, compared to the true network.

**Figure 5:**
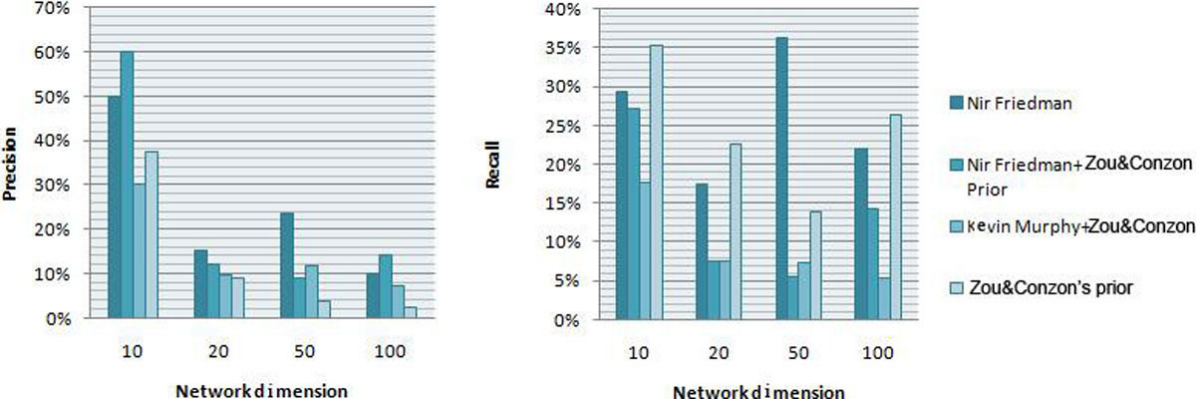
**Comparison of performance between different structure learning algorithms using synthetic dataset**.

The GRN reconstructed by the modified Friedman method (Method 3) without a preprocessed network is a dense network, as given in Figure 4(d). It is noted that the two regulators (YOR383C and YAL051W) were successfully reconstructed and they interact with 24 and 6 target genes, respectively. The GRN reconstructed by the Friedman method has a much higher structure similarity to the true network than Murphy & Zou (Method 2). In Method 4, the preprocessed network also used Friedman method to reduce the search space. The reconstructed GRN is also a sparse network with only one regulator gene identified, as demonstrated in Figure 4(e). It is seen that Zou&Conzen's algorithm can generate a preprocessed network to narrow down the search space, which is meaningful. While it rules out around 86% of the edges from the complete network, it is also a relatively loose rule to retain a large network for the next level learning algorithm. However, when the network size becomes larger, the precision of the preprocessed network (4.0% in the 50-node case and 2.4% in the 100-node case) gradually drops to the random guess precision (4.4% and 1.8%, respectively), as shown in Figure 5.

A complete performance comparison of the four algorithms in terms of precision and recall is given in Figure 5 and the corresponding data given in Table 1, which shows that Friedman's method gives higher precision and recall than the method of Murphy&Zou in all four networks. These results demonstrate that Friedman's method has a great potential in improving the accuracy of GRNs reconstruction.

**Table 1:** Comparison of performance between different structure learning algorithms using synthetic dataset (C_e_: Correctly infered edges; P: Precision; R: Recall)

	Nir Friedman			Nir Friedman + Zou&Conzon			Kevin Murphy + Zou&Conzon			Zou&Conzon		
**Network Size**	**C_e_**	**P**	**R**	**C_e_**	**P**	**R**	**C_e_**	**P**	**R**	**C_e_**	**P**	**R**

10	5	0.50	0.29	3	0.60	0.27	3	0.30	0.18	6	0.38	0.04
												
20	7	0.15	0.17	3	0.12	0.08	3	0.10	0.08	9	0.09	0.23
												
50	38	0.23	0.36	6	0.09	0.06	8	0.12	0.07	14	0.04	0.14
												
100	38	0.10	0.22	25	0.14	0.14	8	0.07	0.05	48	0.02	0.26

### Real yeast benchmark dataset

**Figure 6:**
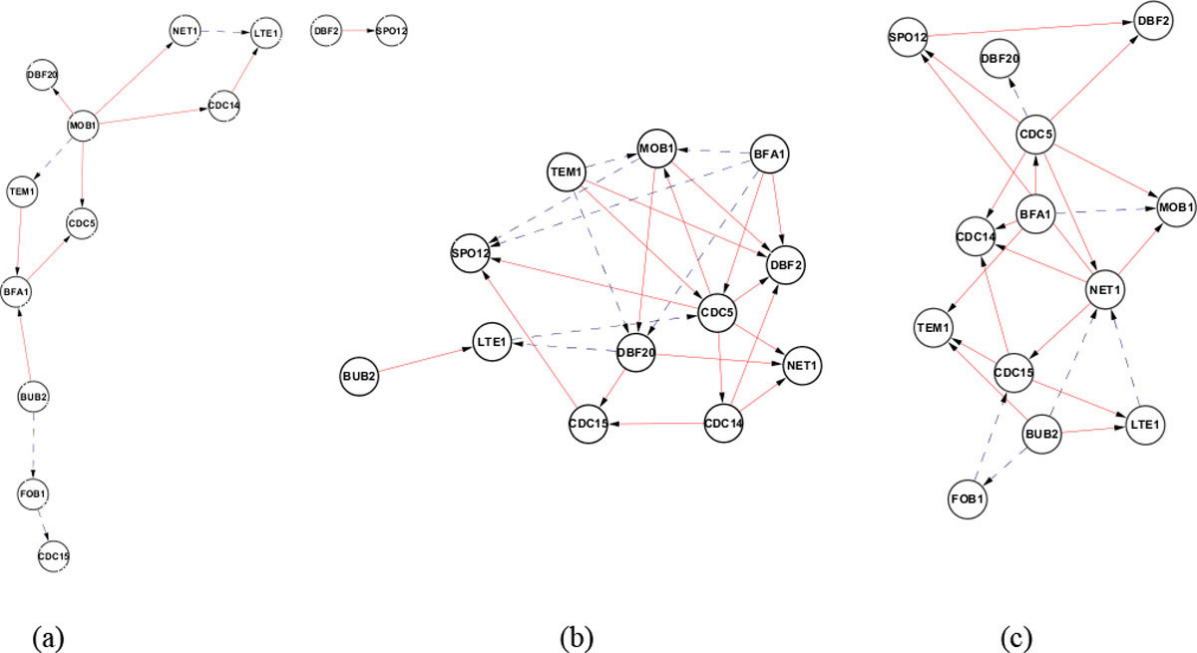
**The real yeast network reconstructed by different algorithms (dashed lines indicating false positive edges, and solid lines true positive edges)**. (a) Murphy + Zou algorithm (b) Probabilistic Boolean Network (c) Friedman's score metrics.

We also investigated the performance of Friedman's DBN algorithm in reconstruct of GRNs from real biological datasets. We tested it on the benchmark yeast time series dataset from Spellman's experiment [19], and compared it with Murphy's DBN algorithm with Zou's preprocessed network [17], as well as a modified Probabilistic Boolean Network algorithm [4]. The dataset is from Spellman's experiment [19], and the interactions are from Saccharomyces Genome Database (SGD) database. The networks reconstructed by these three algorithms are showed as Figure 6 and precision and recall are given in Table 2. The results show that the Friedman's DBN algorithm outperforms Murphy's DBN algorithms in terms of accuracy and recall. Murphy's DBN algorithm shows a sparse network structure, compared with the rest. It is also found that the reconstruction accuracy from real biological datasets (Yeast datasets) is higher than that from the synthetic data.

**Table 2:** Comparison of performance between different structure learning algorithms using yeast benchmark dataset (C_e_: Correctly infered edges; P: Precision; R: Recall)

	Nir Friedman			Kevin Murphy + Zou&Conzon			Probabilistic Boolean Network		
**Network Size**	**C_e_**	**P**	**R**	**C_e_**	**P**	**R**	**C_e_**	**P**	**R**

13	19	0.76	0.19	11	0.69	0.11	20	0.71	0.20

## Conclusions

In this study, we implemented Friedman's score metrics for DBNs by our algorithm, and applied the algorithm in reconstruction GRNs using both synthetic time series gene expression data and a real yeast benchmark dataset. The algorithm is able to capture the correlation between consecutive time slices in both score function and learning procedure, thus Friedman's score metrics gives a higher precision and recall than the naive REVEAL algorithm application in the absence or presence of preprocessed network generated by Zou&Conzen's algorithm. This also reflects that in real biological processes, time lag regulation might better describe the true regulation between genes. Also based on the testing results, the Friedman's score metrics we implemented has great potential in improving the accuracy of structure prediction for GRN reconstruction with complete synthetic time series data.

## Competing interests

The authors declare that they have no competing interests.

## Authors' contributions

HL implemented the algorithms, conducted network inference and performance comparison. HL and CZ drafted the paper. CZ, PG and EJP supervised this work and revised the paper. NW participated in algorithm development and network inference. All authors read and approved the final manuscript.
